# Effectiveness of Continuous Glucose Monitoring in Glucose Control and Quality of Life Among Type 1 Diabetic Patients in Madina City: A Cross-Sectional Study

**DOI:** 10.7759/cureus.65100

**Published:** 2024-07-22

**Authors:** Abdulaziz Altamimi, Nader Moneer Alqeraf

**Affiliations:** 1 Preventive Medicine, Saudi Ministry of Health, Madina, SAU; 2 Health Affairs, Saudi Ministry of Health, Madina, SAU

**Keywords:** type 1 diabetic patients, diabetes, quality of life, glucose control, continuous glucose monitoring

## Abstract

Introduction: Diabetes is a growing global health problem, affecting millions of people worldwide and in Saudi Arabia in particular. Continuous glucose monitoring (CGM) offers real-time glucose levels, alerts, and 24/7 coverage, making it an affordable treatment option. The study aimed to assess CGM's effect on diabetes control and quality of life among type 1 adult diabetic patients in Saudi Arabia.

Methods: This cross-sectional study enrolled Saudi adults diagnosed with type I diabetes and was conducted from 1 April 2024 to 30 May 2024 in Madinah City, Saudi Arabia. Data was collected from patients' medical records and the diabetes quality of life questionnaire (DQoL).

Results: This study enrolled 317 participants, mostly male (52.1%) and a mean age of 34.34±12.28 years. After three months, the HbA1c levels significantly decreased (p<0.001). Older participants reported lower overall quality of life and satisfaction with their level of well-being when using CGM. Univariate analysis found that age had a significant negative association with the total score (B=-0.062, P=0.049) and satisfaction (B=-0.109, P=0.011. Marital status significantly affected the impact score (B=0.567, P=0.024). Education level notably affected satisfaction (B=-0.906, P=0.008) and monthly income influenced satisfaction (B=-1.25, P=0.033). However, multivariate analysis showed that age, education level, and monthly income did not significantly (p>0.005) affect the CGM impact, quality of life, and satisfaction.

Conclusion: These findings indicate that CGM significantly improved diabetes control, while improved quality of life was not significant. The impact, quality of life, and satisfaction were influenced by age, marital status, education, and income level, though not statistically significant independent predictors. Therefore, we recommend longitudinal studies, controlling for confounders.

## Introduction

Diabetes has been on the rise for decades, with 463 million people worldwide living with type 2 diabetes and 1.1 million people under the age of 20 having type 1 diabetes [1]. The International Diabetes Federation (IDF) reports that one in 10 adults have diabetes, but one in two adults with diabetes is undiagnosed [2]. A staggering 10% of global health expenditure is dedicated to the treatment of diabetes, with future projections showing that by 2045, 700 million people will be diagnosed as diabetic [1]. Diabetes may cause several complications if not appropriately controlled, including cardiac disease, stroke, retinopathy that may progress to blindness, kidney failure, and limb amputations resulting from the progression of diabetic foot problems.

Continuous glucose monitoring (CGM) provides information unattainable by intermittent capillary blood glucose, including instantaneous real-time display of glucose level and rate of change of glucose, alerts, and alarms for actual or impending hypo- and hyperglycemia, ''24/7'' coverage, and the ability to characterize glycemic variability [1,3]. The advantage of this method is that it allows for the use of an alarm in the event of hypo- or hyperglycemia, with the patient or parents determining the warning threshold [1,3]. The use of CGM is often related to an improvement in the hemoglobin A1c (HbA1c) level and/or reduction in the risk of hypoglycemia, depending on the baseline features of the patient population [3].

A systematic review by Jiao et al. examined the cost-effectiveness of CGM compared to self-monitoring of blood glucose in type 1 diabetes patients and found that CGM was an economical method, making it a practice treatment option [4]. A clinical trial by Wan et al. concluded that CGM is cost-effective, with improved glucose control and a reduction in severe hypoglycemia episodes [5].

As of June 2020, Saudi Arabia had a population of slightly more than 34.8 million, with an adult diabetes prevalence of 18.3%. The IDF also ranked Saudi Arabia as the seventh-highest country for new cases of type 1 diabetes per year. A study conducted among Saudi patients with type 1 diabetes mellitus showed that patients' views had a substantial influence on their willingness to adopt the technology; the intricacy of new technologies, as well as the lengthy procedure of procuring the CGM device, slowed its adoption. However, the user-friendly interfaces of such devices influenced adoption intentions [6]. Another study showed that patients who checked their glucose levels more regularly experienced considerably fewer complications (p=0.002), and CGM was the most effective monitoring approach, with the lowest rate of problems compared to other methods (p=0.002) [7].

CGM leads to better diabetes control manifested as changes in HbA1c level and quality of life among type 1 diabetic patients [8,9]. Though there is evidence of the effectiveness of CGM, research conducted in Saudi Arabia exploring the use of CGM is still limited despite the high prevalence of diabetes mellitus among the Saudi population. Therefore, the study aimed to assess the effect of CGM on disease control manifested as changes in HbA1c level and quality of life among type 1 adult diabetic patients.

## Materials and methods

Study design and population

This was a cross-sectional study conducted in Madinah, Saudi Arabia, from 1 April 2024 to 30 May 2024, to determine the effectiveness of CGM in glucose control and improving the quality of life among type 1 diabetic adult patients.

This study enrolled Saudi adults diagnosed with type I diabetes mellitus for more than one year, who have been using insulated CGM for at least six months, attending multi-diabetic centers in Madina City, Saudi Arabia.

Non-Saudi patients, newly diagnosed patients, patients with a recent history of steroid or alcohol abuse, patients with a recent history of surgery/severe trauma/infection/hospitalization, and pregnant or lactating patients were all excluded.

The average number of patients with diabetes who attend diabetic centers in Madinah city is around 1500 yearly. This number was used to calculate the sample size needed for this study using the Open Epi sample size calculator. Considering a 95% confidence interval (CI) and a 5% margin of error, the minimum required sample size was calculated to be 306. After adding 10% for the non-response rate, the final sample size was 336. 

Participants were selected using a simple random selection technique from the diabetic centers at King Fahad Hospital (KFH) and King Salman Bin Abdulaziz Medical City (KSAMC), two main healthcare facilities managing type 1 diabetic patients in Madinah city. The two health centers operate in the Madinah province under the supervision of the Ministry of Health. Authors employed by the Ministry of Health have been given access to the electronic systems of both centers.

Data collection tool

Data on demographic characteristics, clinical data, and three readings of HbA1c were collected from patients’ medical records. The mean values of HbA1c at baseline of insulation CGM and the mean values of HbA1c at baseline, three months, and six months of CGM use were collected. This study used the Arabic version of the Diabetes Quality of Life questionnaire (DQoL) to assess the quality of life [10]. The DQoL questionnaire has been used for several years in various countries and languages. The questionnaire is composed of 46 items divided into three domains: satisfaction (15 items), impact (20 items), and worries (11 items divided into social/vocational and diabetes-related). The satisfaction and impact questions include a 5-point Likert scale (very satisfied (5 points), quite satisfied, satisfied, little satisfied, and very dissatisfied (1 point)). Questions related to worries about diabetes are divided into two sections: worries about social/vocational issues and worries about the future effects of diabetes. Responses to these are dichotomous, with Yes or No options. However, having dichotomous and 5-point Likert scales may cause serious issues when attempting to validate questionnaires; therefore, these statements were converted to 5-point Likert scale responses. The Diabetic Quality of Life (DQoL) questionnaire is a widely used tool and has demonstrated strong validity and reliability [11,12]. However, the questionnaire has been translated into different languages including Arabic language and validated [13].

Statistical analysis

Data were entered and organized into a Microsoft Office Excel 2019 datasheet. Then, SPSS version 23 statistical software (SPSS Inc., USA) was used for statistical analysis. Descriptive statistics were performed, and the data were expressed as means with SD. Inferential statistics were performed using the T-test for normally distributed data and the Mann-Whitney test for not normally distributed data. The repeated measured ANOVA was used to test differences in arithmetic means of the three groups (baseline, three, and six months after the insulation of CGM). However, simple and multiple linear regressions were conducted to evaluate factors associated with the DQoL questionnaire's total mean score and the mean scores of each factor. A value of P<0.05 was considered statistically significant, and CI was calculated.

Ethical considerations

Participants were informed of the research purpose and of their role in terms of time and effort. Every individual had full autonomy for participation and free power of choice (voluntariness) to participate. Confidentiality was ensured by the use of the anonymous questionnaire. This study was approved by King Salman bin Abdulaziz Medical City Institutional Review Board (IRB log No: 24-015).

## Results

Demographic characteristics of the study sample

The demographic characteristics of the participants in the study are presented in Table 1. The sample comprised 317 individuals, with a slight predominance of males (52.1%) over females (47.9%). The majority of the participants were married (62.8%) and held a bachelor's degree (47.3%). The distribution of monthly income showed that 51.4% of the participants earned between 5000 and 10000 SAR, followed by 36.3% who earned less than 5000 SAR. The mean age of the participants was 34.34±12.28 years, and they had been living with the disease for an average of 12.28±7.52 years.

**Table 1:** Demographic information of the participants in the study

		Frequency	Percent
Gender	Female	152	47.9
	Male	165	52.1
Marital status	Not married	97	30.6
	Married	199	62.8
	Divorced	15	4.7
	Widower	6	1.9
Education level	Secondary	129	40.7
	Bachelor	150	47.3
	Post-graduate	38	12.0
Monthly income	Less than 5000 SAR	115	36.3
	5000-10000 SAR	163	51.4
	More than 10000 SAR	39	12.3
Age (Years)	Mean ± SD	34.34±8.98	
Diabetic disease duration (years)	Mean ± SD	12.28±7.52	

Levels of quality-of-life (satisfaction, impact, and worry subscales) among participants

Table 2 shows the levels of satisfaction with the level of well-being, impact, worry, and total scores among participants using CGM for at least six months. The satisfaction score ranged from 23 to 70, with a mean score of 40.62±6.835, indicating moderate satisfaction levels. The impact score, out of a possible 55, had a range from 11 to 49, with a mean of 35.20±5.043, suggesting a significant impact on participants' lives. The worry score, which could reach up to 20, varied between four and 19, with a mean score of 12.31±2.267, reflecting a moderate level of concern among the participants. The total scores, combining all these aspects with a possible maximum of 145, ranged from 75 to 112, with a mean score of 88.13±5.03.

**Table 2:** Descriptive statistics of the study scales

	Total possible score	Minimum	Maximum	Mean	SD
Satisfaction	70	23	70	40.62	6.835
Impact	55	11	49	35.20	5.043
Worry	20	4	19	12.31	2.267
Total scores	145	75	112	88.13	5.03

HbA1c levels of the study sample

Table 3 shows the HbA1c levels among participants at baseline, three months, and six months after using CGM. The baseline HbA1c levels ranged from 5.6% to 13.7%, with a mean of 8.79%±1.63%, indicating relatively high initial glucose levels among the participants. After three months, the HbA1c levels decreased, ranging from 5.1% to 12.6%, with a mean of 8.24%±1.30%. This downward trend significantly (p<0.001) continued for six months, indicating a significant improvement in glucose control among the participants over six months and highlighting the effectiveness of CGM in managing HbA1c levels.

**Table 3:** Descriptive statistics of the HbA1c measurements in the study The P-value is calculated by a repeated measured ANOVA. **Significant at P-value <0.01

	Minimum	Maximum	Mean	SD	P-value
HbA1c at baseline	5.6	13.7	8.79	1.63	<0.001**
HbA1c at 3 months	5.1	12.6	8.24	1.30	
HbA1c at 6 months	5.1	14.6	7.78	1.39	

Factors affecting the quality-of-life scores (the satisfaction, impact, and worry subscales) among CGM users

Table 4 presents the simple linear regression analysis results for various factors affecting the scores of the DQoL, examining the total score as well as the satisfaction, impact, and worry subscales among participants using CGM for at least six months. Age had a significant negative association with the total score (B=-0.062, P=0.049) and the satisfaction subscale (B=-0.109, P=0.011), indicating that older participants reported lower overall quality of life and satisfaction. Marital status significantly affected the impact score (B=0.567, P=0.024). Education level had a notable effect on satisfaction (B=-0.906, P=0.008) and worry (B=0.244, P=0.031), suggesting that higher education was associated with lower satisfaction but increased worry. High monthly income (B=-1.25, P=0.033) was associated with lower satisfaction levels. 

**Table 4:** Simple linear regression analysis for different factors affecting the scores of DQoL *Significant at P-value <0.05. **Significant at P-value <0.01 DQoL, diabetes Quality of Life Questionnaire

Variables	Mean total score			Satisfaction			Impact			Worry		
	B	P-value	95% CI	B	P-value	95% CI	B	P-value	95% CI	B	P-value	95% CI
Gender	1.051	0.063	-0.058-2.161	0.575	0.455	-0.938-2.088	0.287	0.614	-0.830- 1.403	0.189	0.458	-0.312-0.691
Age	-0.062	0.049*	-0.124-0.000	-0.109	0.011*	-0.193- -0.026	0.200	0.664	-0.705-1.105	-0.001	0.932	-0.029-0.027
Marital status	-0.640	0.163	-1.541-0.261	-0.904	0.147	-2.126-0.319	0.567	0.024*	0.075-1.059	0.063	0.759	-0.343-0.470
Education level	-0.095	0.705	-0.590-0.400	-0.906	0.008**	-1.571- -0.241	0.805	0.063	-0.043-1.653	0.244	0.031*	0.023-0.466
Monthly income	-0.239	0.581	-1.090-0.612	-1.25	0.033*	-2.39- -0.099	-0.001	0.973	-0.076-0.073	0.202	0.299	-0.180-0.585
Diseases years	-0.028	0.456	-0.102-0.046	0.002	0.964	-0.098-0.103	0.035	0.841	-0.308-0.377	-0.029	0.086	-0.062-0.004
HbA1c at baseline	-0.296	0.088	-0.636-0.045	-0.279	0.237	-0.742-0.184	0.105	0.633	-0.326-0.536	-0.052	0.507	-0.206-0.102
HbA1c at 3 months	-0.103	0.639	-0.533-0.328	-0.241	0.417	-0.825-0.343	0.045	0.825	-0.356-0.446	0.034	0.732	-0.160-0.228
HbA1c at 6 months	-0.095	0.641	-0.495-0.305	-0.142	0.608	-0.686-0.402	0.287	0.614	-0.830-1.403	0.002	0.984	-0.178-0.182

Table 5 presents the results of a multivariate analysis of significant factors affecting the satisfaction scores among the participants using CGM for at least six months. Age showed a negative association with satisfaction (B=-0.079, (95%CI: -0.183 to 0.025, P=0.138), indicating that older participants tended to report lower satisfaction levels, although this relationship was not statistically significant. Education level also demonstrated a negative association with satisfaction (B=-1.103, P=0.122) suggesting that higher educational attainment was linked to lower satisfaction, but this effect did not reach statistical significance (CI: -2.502 to 0.297). Monthly income had a positive but non-significant association with satisfaction (B=1.069, P=0.400) indicating that higher income may be related to increased satisfaction; however, this was not statistically supported (CI: -1.428 to 3.565). 

**Table 5:** Multivariate analysis for significant factors affecting the scores of satisfaction

Variables	B	P-value	95% CI	
			Lower bound	Upper bound
Age	-0.079	0.138	-0.183	0.025
Education level	-1.103	0.122	-2.502	0.297
Monthly income	1.069	0.400	-1.428	3.565

## Discussion

The findings showed the significant impact of CGM on improving glucose control among patients with type 1 diabetes, aligning with previous studies conducted in other countries [7,10,14]. The consistent decrease in HbA1c levels over six months demonstrates the long-term effectiveness of this method in managing diabetes. Our findings are consistent with previous research showing the effectiveness of CGM in improving glucose control, as shown by HbA1c. Lind et al. also showed that CGM, compared to traditional treatment for 26 weeks, resulted in decreased HbA1c in patients with poorly controlled type 1 diabetes treated with several daily insulin injections [14]. A previous study found that CGM was associated with a significant reduction in HbA1c levels compared to self-monitoring of blood glucose [15]. Another study comparing CGM and self-monitoring of glucose found that the use of CGM increased life expectancy by 1.32 years and QALYs by 1.63, compared to self-monitoring [16].

Our findings confirm that CGM is associated with an improved quality of life among patients with type 1 diabetes mellitus. This might be explained by the ease of use, user-friendliness of CGM, and reduction of pain usually associated with multiple injections endured during self-monitoring of glucose. Tanaka et al. assessed the emotional anguish among diabetes patients who self-monitored their blood glucose and found that patients who reported pain due to self-monitoring had higher mental distress, lower health-related quality of life, and higher glycated hemoglobin [17]. Self-monitoring was less appreciated by patients experiencing pain and those with regular and daily glucose check-ups in clinical settings. However, our study showed that the impact of CGM on quality of life is influenced by various demographic factors, such as age, marital status, education level, and monthly income. Older participants and those with higher education levels reported lower satisfaction levels. This could be due to the fact that older individuals may have more complex health needs and may face additional challenges in managing their diabetes. This might explain the previous study's findings, which found that CGM use was higher among young patients than older patients. However, adolescent patients continued to have the lowest usage rates of CGM [9]. Those with higher education levels may have higher expectations or a better understanding of the potential benefits of CGM, leading to lower satisfaction if their expectations are not fully met. There is also evading suggesting that diabetes education intervention leads to a significant reduction in average HbA1c levels after three months (p=0.001) [18]. This may be attributed to the improved knowledge of CGM device use among those educated, as it was found that lag time and calibrations were the most difficult, needing education among CGM device users [19]. The finding that married participants experienced a greater impact of CGM on their quality of life could be attributed to the support and involvement of their spouse in managing the disease, which may amplify the impact of CGM. Studies showed that CGM may improve collaborative diabetes treatment, and its impact is influenced by marital relationships among type 1 diabetes patients and their spouses [20]. Monthly income negatively influenced satisfaction with CGM among participants, consistent with previous studies that showed a relationship between CGM use and financial constraints, mainly due to associated costs [9,21]. However, though there are upfront CGM device costs, a clinical trial by Wan et al. showed that CGM is cost-effective at the willingness-to-pay threshold of $100,000 per quality-adjusted life year (QALY) for persons with type 1 diabetes mellitus compared to multiple insulin injections, which still lead inadequate glycemic control, while CGM improved glucose control [5]. 

That univariate analysis showed that age, marital status, education level, and monthly income significantly influenced the impact, quality of life, and satisfaction with CGM among participants; the multivariate analysis did not show any statistically significant association between those demographics and quality of life and satisfaction. This indicates that changes observed in HbA1c levels and quality of life might also be due to other confounding factors like modifications in treatment regimen or lifestyle changes and others, highlighting the need for further studies with designs capable of establishing causal relationships to be certain.

One of the major limitations of this study is its cross-sectional design, which does not allow for the determination of cause-and-effect relationships between variables. The use of self-reported data collected through the DQoL questionnaire is another limitation, as respondents might not have accurately remembered or reported their experiences. The absence of a control group is another limitation as such; it becomes difficult to ascertain whether changes observed in HbA1c levels and quality of life were exclusively due to CGM or rather instigated by other factors like modifications in treatment regimen or lifestyle changes. Therefore, we recommend that future researchers address these limitations by conducting longitudinal studies involving control groups and employing both subjective and objective methods to evaluate diabetes management efficiency through CGM. Moreover, there is a need for qualitative studies that will give more insights into how different demographic factors shape peoples’ experience while using CGM for managing type 1 diabetes.

## Conclusions

The study found that CGM positively significantly impacts glucose control, with a decrease in HbA1c levels over six months, while improved quality of life was not significant. However, the impact is moderated by demographic factors such as age, marital status, education level, and monthly income. Age is the most important factor in analyzing the effect of CGM on quality of life, with older individuals and those with higher education reporting lower satisfaction levels. Married individuals experienced greater effects, while higher incomes were associated with lower satisfaction ratings. The findings support previous research showing the effectiveness of CGM in improving glucose control in diabetic patients. However, future studies should focus on longitudinal designs, controlling for confounders and using both subjective and objective measurements. Moreover, implementing CGM methods for managing type 1 diabetes requires careful implementation, ensuring targeted interventions to ensure improved quality of life.

## References

[1] Saeedi P, Petersohn I, Salpea P (2019). Global and regional diabetes prevalence estimates for 2019 and projections for 2030 and 2045: Results from the International Diabetes Federation Diabetes Atlas, 9(th) edition. Diabetes Res Clin Pract.

[2] (2024). IDF: IDF Diabetes Atlas. https://diabetesatlas.org.

[3] Rodbard D (2016). Continuous glucose monitoring: a review of successes, challenges, and opportunities. Diabetes Technol Ther.

[4] Jiao Y, Lin R, Hua X (2022). A systematic review: cost-effectiveness of continuous glucose monitoring compared to self-monitoring of blood glucose in type 1 diabetes. Endocrinol Diabetes Metab.

[5] Wan W, Skandari MR, Minc A (2018). Cost-effectiveness of continuous glucose monitoring for adults with type 1 diabetes compared with self-monitoring of blood glucose: the Diamond randomized trial. Diabetes Care.

[6] Ziemba E, Chmielarz W, Wątróbski J (2024). A quantitative and qualitative exploration of critical factors in the IAI-CGM framework: the perspective of Saudi patients with Type 1 diabetes mellitus.

[7] Alkhatieb MT, Aljehani KM, Alkhalifah HA, Alghamdi NS, Almaghrabi AS, Alqarni BB, Alzahrani AY (2023). The impact of frequent glucose monitoring on the prevalence of complications among patients with diabetes in Saudi Arabia. Cureus.

[8] Rachmiel M, Landau Z, Boaz M (2015). The use of continuous glucose monitoring systems in a pediatric population with type 1 diabetes mellitus in real-life settings: the AWeSoMe Study Group experience. Acta Diabetol.

[9] DeSalvo DJ, Miller KM, Hermann JM (2018). Continuous glucose monitoring and glycemic control among youth with type 1 diabetes: international comparison from the T1D Exchange and DPV Initiative. Pediatr Diabetes.

[10] Bujang MA, Adnan TH, Mohd Hatta NK, Ismail M, Lim CJ (2018). A revised version of diabetes quality of life instrument maintaining domains for satisfaction, impact, and worry. J Diabetes Res.

[11] The DCCT Research Group (1988). Reliability and validity of a diabetes quality-of-life measure for the diabetes control and complications trial (DCCT). Diabetes Care.

[12] Diabetes Control and Complications Trial Research Group (1996). Influence of intensive diabetes treatment on quality-of-life outcomes in the diabetes control and complications trial. Diabetes Care.

[13] Al-Qerem W, Al-Maayah B, Ling J (2021). Developing and validating the Arabic version of the Diabetes Quality of Life questionnaire. East Mediterr Health J.

[14] Lind M, Polonsky W, Hirsch IB (2017). Continuous glucose monitoring vs conventional therapy for glycemic control in adults with type 1 diabetes treated with multiple daily insulin injections: the gold randomized clinical trial. J Am Med Assoc.

[15] Chehregosha H, Khamseh ME, Malek M, Hosseinpanah F, Ismail-Beigi F (2019). A View Beyond HbA1c: Role of Continuous Glucose Monitoring. Diabetes Ther.

[16] Molaee MC, Naseri ZG, Karami MA (2024). Long-term cost-effectiveness of continuous glucose monitoring versus self-monitoring of blood glucose in adults with type 1 diabetes in Iran. Value Health Reg Issues.

[17] Tanaka N, Yabe D, Murotani K (2018). Mental distress and health-related quality of life among type 1 and type 2 diabetes patients using self-monitoring of blood glucose: a cross-sectional questionnaire study in Japan. J Diabetes Investig.

[18] Borges LP, de Jesus PC, de Souza JB (2024). The impact of diabetes education on continuous glucose monitoring in SUS-dependent patients in a northeastern Brazilian city. Life (Basel).

[19] Messer L, Ruedy K, Xing D, Coffey J, Englert K, Caswell K, Ives B (2009). Educating families on real time continuous glucose monitoring: the DirecNet navigator pilot study experience. Diabetes Educ.

[20] Ritholz MD, Beste M, Edwards SS, Beverly EA, Atakov-Castillo A, Wolpert HA (2014). Impact of continuous glucose monitoring on diabetes management and marital relationships of adults with type 1 diabetes and their spouses: a qualitative study. Diabet Med.

[21] Karakuş KE, Sakarya S, Yeşiltepe Mutlu G (2021). Benefits and drawbacks of continuous glucose monitoring (CGM) use in young children with type 1 diabetes: a qualitative study from a country where the CGM is not reimbursed. J Patient Exp.

